# Relationship Between Population Size and Habitat Area of Giant Pandas in China

**DOI:** 10.3390/ani15020117

**Published:** 2025-01-07

**Authors:** Dongwei Kang

**Affiliations:** School of Ecology and Nature Conservation, Beijing Forestry University, Beijing 100083, China; kangdw@bjfu.edu.cn

**Keywords:** county, giant panda survey, habitat, nature reserve, population

## Abstract

This study analyzed survey result data of giant panda populations and habitats in the third and fourth giant panda surveys to reveal the population size–habitat area relationship and assess the habitat status at the county and nature reserve levels. Results showed that a significant linear relationship existed between the logarithm of population size (lnS) and the logarithm of habitat area (lnA), with an equation of lnS = 1.145 × lnA − 4.022. Based on this relationship, this study determined that 557.43 km^2^ was the minimum habitat area for maintaining a sustainable survival population of 25 pandas (the minimum population size to ensure sustainable survival with a relatively low extinction probability). This study selected 20 counties and 16 nature reserves containing ≥25 pandas and found that 30.0% of counties and 87.5% of reserves had habitat areas less than 557.43 km^2^. More habitat restoration efforts are needed to increase the habitat. The habitat area remained stable in the selected counties but decreased in the selected reserves between the third and fourth surveys. The causes of habitat decline in counties and reserves should be identified so that targeted conservation measures can be implemented. This study provides a theoretical basis and scientific reference for giant panda conservation and habitat management.

## Simple Summary

This study analyzed the population-habitat relationship of giant pandas (*Ailuropoda melanoleuca*), and found that 557.43 km^2^ was the minimum habitat area needed for a sustainable population with 25 pandas. Based on this criterion, this study evaluated the habitat status of 20 counties and 16 nature reserves containing ≥25 pandas. It was observed that 30.0% of counties and 87.5% of reserves had habitat areas less than 557.43 km^2^, indicating that more restoration efforts are required to increase the habitat area. In some counties and reserves with habitat areas ≥557.43 km^2^, the habitat had obviously decreased between the third (conducted from 1999 to 2003; the population size was 1596, and the habitat area was 2,304,991 ha) and fourth (conducted from 2011 to 2014; the population size was 1864, and the habitat area was 2,576,595 ha) giant panda surveys. The specific causes should be identified so that targeted conservation measures can be implemented.

## 1. Introduction

The giant panda (*Ailuropoda melanoleuca*) is a rare animal that lives in bamboo forests. Its survival status has attracted much attention. Many measures have been implemented to protect this species, such as the establishment of protected areas [1], efforts of major ecological projects [2], and scientific conservation studies [3]. Together, these measures have resulted in significant progress. For example, the extinction risk of the giant panda has been downgraded from endangered to vulnerable [4,5], although this downgrade may have been premature [6,7].

Giant pandas had a much wider distribution historically [8]. However, this species is currently only found in Sichuan, Shaanxi, and Gansu provinces [9]. Due to the influence of natural factors, such as earthquakes [10] and landslides [11], and human activities, such as grazing [12], road construction [13], tourism [14], and logging [15], 33 local populations of the giant panda exist [9].

Analyzing the population–habitat relationship provides key information for giant panda protection. Many studies have emphasized the importance of protecting small populations [16,17,18,19,20]. The status of the main populations cannot be ignored. Previous achievements may be lost if the habitat conditions of the main populations cannot be guaranteed and maintained. However, many fundamental questions in this regard have not been resolved, limiting the formulation and implementation of conservation strategies. For example, it is unknown how much habitat area is required to maintain the survival of a sustainable population. It is critical to conduct relevant studies to address this research gap.

Sufficient and adequate habitat is vital to the survival of giant pandas [20,21]; however, analyzing the habitat dynamics is challenging. China has conducted four national surveys to monitor the giant panda and its habitat [9,11,16,22]. Information on the population structure and habitat pattern of giant pandas obtained from the fourth survey has been released [9]. After each survey, information on the number of giant pandas and habitat area by taking the units of county and nature reserve are usually available [9,11,16]. These resources enable an analysis of the population–habitat relationship and assess habitat area changes.

This study analyzed the survey result data on the population and habitat obtained from the third and fourth giant panda surveys. The objectives were to (1) determine the relationship between the population size and habitat area and (2) describe the habitat area and changes in critical distribution areas of the giant panda. This study quantified the habitat area required for maintaining the sustainable survival of a population with adequate individuals and assessed the habitat area status at the county and nature reserve levels. The goal was to provide a theoretical basis and scientific reference for giant panda conservation and habitat management.

## 2. Methods

### 2.1. Data Sources

The third survey was conducted from 1999 to 2003 and showed that 1596 giant pandas lived in an area covering 2,304,991 ha [11]. The fourth survey was conducted from 2011 to 2014, indicating that 1864 giant pandas lived in a habitat covering 2,576,595 ha [9]. The fourth survey partly continued the technology and methods used in the third survey, making some results comparable [9]. For example, transect surveys were used to determine the giant panda distribution, and the traveling distance and bamboo stem fragment distinguishing methods were used to obtain population statistics. Giant panda habitats must meet two conditions. There must be a distribution of giant pandas, and the habitat conditions must be suitable for survival and reproduction, i.e., the habitat must include forests and bamboo [9,11]. The surveys used the same methods to determine the habitat area. Ecological factors critical to giant pandas were considered, such as their activities and habits, vegetation status, bamboo distribution, elevation, human disturbance, and landscape factors. The details of the survey and analysis methods have been published [9,11].

This study used the survey result data on population and habitat from the fourth survey, including the names, sizes, and habitat areas of the local populations [9] (Table S1). Specific conservation work on the giant panda and its habitat is mainly carried out at the county and nature reserve levels, and the survey result data at these two levels are available in the third and fourth surveys. This study utilized the number of giant pandas and the habitat area as indicators because they were available and comparable between the two surveys.

### 2.2. Data Analysis

This study analyzed the relationship between the population size and habitat area of 33 local populations and obtained trends using scatter plots [23]. If data were scattered or the trend was unclear, the data were transformed (such as logarithmic transformation) [24]. This study assessed the relationship (e.g., its linearity) using the determination coefficient (R^2^), analysis of variance (ANOVA), standardized residual, and significance level [25].

A giant panda population requires a certain number of individuals to ensure survival. For example, under the circumstances of no significant environmental changes occurring and the survival ability of individuals in the population being unchanged, if surviving for 200 years with an extinction probability <0.05 is adopted as the standard for maintaining the sustainable survival of a giant panda population, the minimum population size is 28–30. When the extinction probability is 0.076, the minimum population size is 25 [26]. This study assumed 25 as the minimum population size of a sustainable survival population. The extinction probability was <0.10. This number provided a sufficient sample size. Finally, based on the determined relationship between population size and habitat area, this study calculated the habitat area required to ensure the sustainable survival of a population with 25 pandas.

The fourth survey indicated that giant pandas appeared in 49 counties, and 67 nature reserves were established to protect them [9]. This study identified the counties and nature reserves containing ≥25 pandas to describe the habitat area and changes in the fourth survey. For the selected counties and reserves, this study examined if habitat areas were sufficient to ensure the panda’s sustainable survival for a minimum population size. The habitat area, number of giant pandas in the selected counties and reserves, and their proportions nationwide and in the giant panda nature reserve system were calculated. This study collected data on the habitat area of the corresponding counties and reserves from the third survey. The results were compared with the data from the third and fourth surveys to assess habitat changes. Paired-sample *t*-tests or non-parametric tests of two related samples were used based on statistical requirements [23]. This study also compared the change rate of the habitat area in the counties and reserves between the third and fourth surveys by dividing the difference in the parameter values between the fourth and third surveys by the parameter value of the third survey and expressing it as a percentage. To facilitate description, this study focused on counties and reserves that did not meet the minimum habitat area requirement and/or exhibited a significant decrease in the habitat area (i.e., >10%). Furthermore, this study did not ignore the habitat area in counties and reserves that contained <25 pandas in the fourth survey.

## 3. Results

### 3.1. Relationship Between Population Size and Habitat Area of Giant Pandas

**Figure 1:**
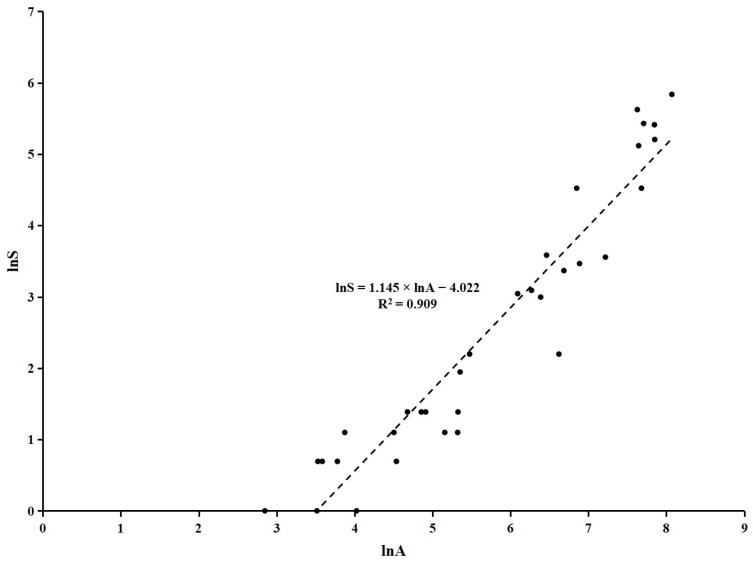
The scatter plot of the logarithm of population size (lnS) (logarithmic transformation of the population size) and the logarithm of habitat area (lnA) (logarithmic transformation of the habitat area) of giant pandas.

The population size and habitat area for the 33 giant panda local populations had a large range. The population size ranged from 1 to 343, and the habitat area ranged from 17.27 km^2^ to 3199.26 km^2^ (Table S1). This study performed logarithmic transformation on both datasets of population size and habitat area to facilitate data centralization. A linear trend was observed between the logarithm of population size (lnS) and the logarithm of habitat area (lnA) (Figure 1).

The linear model describing the relationship between lnS and lnA had an R^2^ value of 0.909, indicating a high fitting degree. The *p*-value of the ANOVA was <0.001 (F = 310.318), and the standardized residual ranged from −2.395 to 1.611. The *p*-values of lnA and the constant in the model were <0.001 (Table 1). Therefore, the linear relationship between lnS and lnA was significant. The equation was lnS = 1.145 × lnA − 4.022 (Figure 1, Table 1). Based on this equation, A = 557.43 km^2^ when S = 25, i.e., a habitat area of 557.43 km^2^ was required to ensure the sustainable survival of a population with 25 pandas.

### 3.2. Habitat Area and Changes in Main Distribution Counties

An analysis of the 49 counties in the fourth survey indicated that 40.8% (20 of 49) contained ≥25 pandas. Among the 20 counties, 30.0% (6 of 20) had a habitat area of <557.43 km^2^: Chongzhou, Dayi, Maoxian, Qingchuan, Yangxian, and Lushan (Table S2). Among the 29 counties containing <25 pandas, one county (Ningshan) had a habitat area of >557.43 km^2^ (609.40 km^2^). Since only 23 giant pandas were in Ningshan, this area was not included in the analysis.

**Figure 2:**
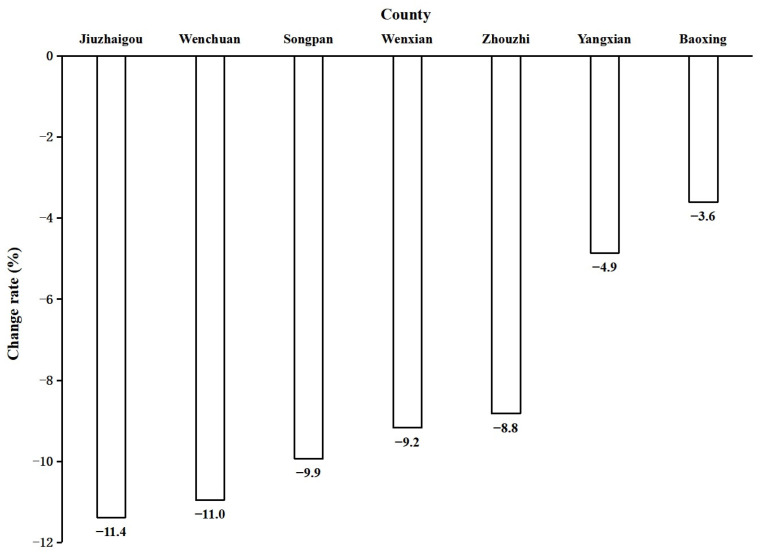
Change rate of habitat area in the counties with a habitat decrease from the third (1999–2003) to the fourth (2011–2014) giant panda survey.

In the fourth survey, the habitat area and number of giant pandas in the 20 counties were 19,148.07 km^2^ and 1671, accounting for 74.3% (19,148.07 of 25,765.95) of the national habitat area and 89.6% (1671 of 1864) of the national population size, respectively. The mean habitat area increased from 913.74 km^2^ to 957.40 km^2^ from the third to the fourth survey, but the area increase was not significant (*p* > 0.05; Table 2). The habitat area of 35.0% (7 of 20) of the counties decreased between the two surveys. The habitat area of Yangxian, which was <557.43 km^2^, decreased by 4.9%, and among the six counties with values ≥557.43 km^2^, the habitat area of Jiuzhaigou and Wenchuan decreased by >10% (Figure 2; Table S2).

### 3.3. Habitat Area and Changes in Important Nature Reserves

Among the 67 nature reserves in the fourth survey, 23.9% (16 of 67) contained ≥25 pandas. Among the 16 reserves, 87.5% (14 of 16) were <557.43 km^2^, and only 2 were ≥557.43 km^2^ (Wolong and Baishuijiang) (Table S3). None of the 51 reserves containing <25 pandas had habitat areas ≥ 557.43 km^2^.

In the fourth survey, the habitat area and number of giant pandas in the 16 reserves were 6358.91 km^2^ and 888, accounting for 45.9% (6358.91 of 13,851.96) of the habitat area and 71.3% (888 of 1246) of giant pandas in all giant panda nature reserves, respectively. Two reserves (Heizhugou and Huangbaiyuan) were excluded from the further analysis because they had no habitat area data in the third survey.

**Figure 3:**
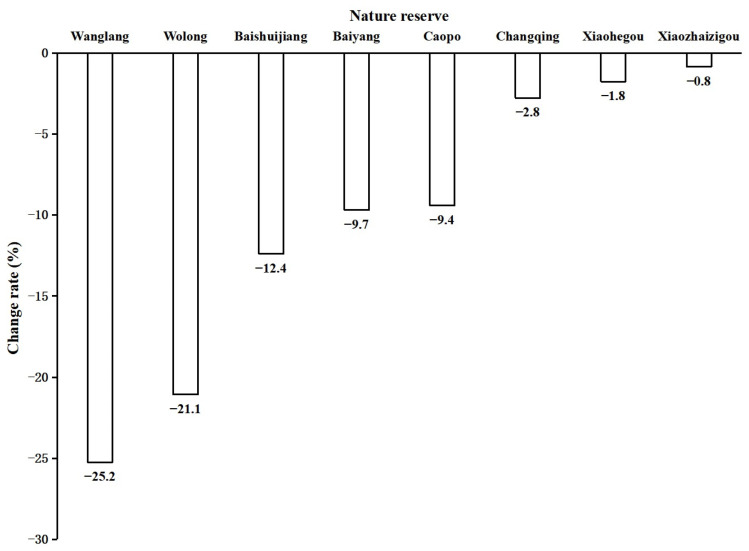
Change rate of habitat area in the nature reserves with a habitat decrease from the third (1999–2003) to the fourth (2011–2014) giant panda survey.

The mean habitat area decreased from 435.80 km^2^ to 417.62 km^2^ from the third to the fourth survey, but this decrease was not significant (*p* > 0.05; Table 2). The habitat area of 57.1% (8 of 14) of the reserves decreased between the two surveys. Regarding the decline rate of the six reserves with a habitat value <557.43 km^2^, the decline rate of Wanglang was >20%, and regarding the two reserves with a habitat value ≥557.43 km^2^, the decline rate of Baishuijiang was >10%, and that of Wolong was >20% (Figure 3; Table S3).

## 4. Discussion

### 4.1. Habitat Area Required for Population Survival

Habitat refers to the environment where an organism or a population lives [27]. Analyzing the population–habitat relationship provides insights into species protection and habitat management. Some previous studies have discussed the population viability of giant pandas [28,29,30,31,32,33], whereas few have focused on the habitat requirements to ensure species survival. This study used survey result data on the population size and habitat area of 33 local giant panda populations from the fourth survey. This is the first study to confirm a linear relationship between lnS and lnA. Importantly, this study found that a habitat area of 557.43 km^2^ was required to ensure the sustainable survival of a population with 25 pandas. By comparing with previous studies, this finding advances the knowledge of the habitat requirements of the giant panda.

An analysis of the 49 counties and 67 reserves in the fourth survey showed that <50% of the counties and <25% of the reserves contained ≥25 pandas. This result indicates that many counties and most reserves do not have the minimum population size required for the species’ sustainable survival. Among the 20 counties and 16 reserves containing ≥25 pandas, 6 counties and 14 reserves did not have a habitat area of 557.43 km^2^, demonstrating that they cannot ensure the sustainable survival of the local giant pandas. Furthermore, many habitats in the counties or reserves may be fragmented and isolated. Although they contain >25 pandas, reaching the minimum area does not necessarily guarantee the sustainable survival of local giant pandas.

A population is a group of individuals of the same species within a given temporal and spatial range [34]. Notably, different individuals in a county or reserve may not belong to the same population. This study considered all individuals in a county or reserve to facilitate analysis. However, the population structure may be more complex. Nevertheless, the minimum habitat area of 557.43 km^2^ obtained in this study has practical significance. It provides a scientific basis for delineating areas for habitat protection and restoration and establishing protected areas.

### 4.2. Habitat Area at the County Level

Evaluating whether protection is required is critical in giant panda research [35,36]. This study selected 20 counties containing ≥25 pandas. They included >80% of the giant pandas and >70% of the habitats in the fourth survey; thus, they represent critical giant panda habitats that deserve attention.

Examining habitat dynamics provides crucial information for analyzing the habitat status [37]. The habitat area in the 20 counties increased from the third to the fourth survey, but this increase was not significant, indicating that the habitat is stable. However, a stable habitat does not necessarily mean that no protection is required. A detailed analysis of each county helps to identify potential protection requirements. The habitat area of five out of six counties with habitat areas smaller than 557.43 km^2^ in the fourth survey increased, whereas that of the Yangxian decreased. None of the counties had sufficient habitat to ensure the sustainable survival of local giant pandas. Therefore, more habitat restoration measures are required to enlarge the habitat range.

Giant panda populations, the habitat area, and the distribution are not static [9,11,38,39,40], and fluctuations are normal. However, the causes of significant reductions in these parameters should be identified, and countermeasures should be implemented in time. The rate of decline of counties with a habitat area >557.43 km^2^ was >10% in some counties, such as Wenchuan and Jiuzhaigou. The focus should be on these areas to ensure giant panda protection.

The Wenchuan earthquake occurred in 2008, damaging large areas of important ecosystems and critical giant panda habitats [41,42,43,44]. The large decline in the habitat area in Wenchuan may be related to the impacts of this earthquake. The time between the Wenchuan earthquake and the fourth survey was short, and many damaged habitats have not yet been restored. Habitat restoration is a long-term process [45,46], and continued efforts are required to restore the damaged habitats.

A large bamboo area bloomed in some areas of Jiuzhaigou in 2005 [18]. The decline in the habitat area in Jiuzhaigou may be related to this phenomenon. Furthermore, significant human activities occur in the giant panda habitats of Jiuzhaigou and Wenchuan [18], causing disturbances. It is necessary to conduct studies to determine the specific reasons for habitat decline to establish targeted habitat restoration and human disturbance control measures.

### 4.3. Habitat Area at the Nature Reserve Level

China has established a large amount of protected areas to protect giant pandas [18]. This study selected 16 nature reserves containing ≥25 pandas. They included >70% of giant pandas and >40% of habitats of all giant panda nature reserves in the fourth survey. Since they represent critical giant panda habitats, these reserves require attention.

Nature reserves had the strictest protection of giant pandas before the establishment of national parks. The habitat area in the 14 nature reserves decreased under strict protection; the protection effect is worrying. The decline rate of the habitat area was >10% in some reserves, such as Wanglang, Wolong, and Baishuijiang. These changes may be related to grazing in Wanglang [47], the Wenchuan earthquake in Wolong [48], and the scope adjustment in Baishuijiang [49]. Livestock grazing is common in Wanglang and is the most common disturbance type in the giant panda habitat [9]. Many studies have investigated the effect of grazing on different types of ecosystems [50,51,52]. In addition to the Wenchuan earthquake, the Songpan-Pingwu and Lushan earthquakes have affected the giant panda habitat [10,53,54]. It is necessary to confirm the reason for the habitat decline so that targeted countermeasures can be implemented.

Nature reserves should protect wild animals. The number of giant panda nature reserves is 67 [9], and >76% of the reserves contained <25 pandas, indicating that the number of giant pandas in most reserves is not large. Fourteen among the sixteen reserves containing ≥25 pandas had a habitat area of less than 557.43 km^2^, indicating that the habitat area was insufficient to ensure the sustainable survival of the local giant pandas. Although two reserves (Wolong and Baishuijiang) were larger than 557.43 km^2^, the habitat area declined between the two surveys. More habitat protection and restoration efforts are required to improve giant panda protection, including but not limited to these 16 reserves.

Many of the 67 reserves are in close proximity [9]. The location and layout of many reserves should be adjusted according to the relationship between populations and habitats to improve species protection [55]. As a new type of protected area, the Giant Panda National Park was established in 2021, which has the highest protection intensity and is expected to break the restrictions of protection models by taking administrative division and nature reserves as a unit. The park provides a chance to carry out giant panda protection with local populations and habitat patches as the core. However, the populations and habitats of the giant pandas in the Liangshan mountains were not included in the park [56]. They should not be treated differently.

### 4.4. Research Limitations and Considerations

This study has some limitations that need to be disclosed. First, the habitat area required to ensure the sustainable survival of a panda population of 25 pandas (557.43 km^2^) was not obtained through long-term observations but calculated using the survey result data of 33 giant panda local populations in the fourth survey. Therefore, verification is required. Second, this study analyzed the habitat area and changes in the main distribution counties and important reserves with giant pandas. However, the counties and reserves were determined by humans and may not be adequate for giant pandas. A better approach may be to track local populations to determine changes in giant panda local populations and their habitats. Third, the giant pandas in a county or reserve do not necessarily belong to the same population, and the habitat may be fragmented. Regardless of the fluctuations in the habitat area, tracking the giant panda populations and habitats using patrols and monitoring is required in counties or reserves with a habitat area of >557.43 km^2^.

Few studies have investigated giant panda habitat restoration [57]. More restoration measures are needed to increase giant panda habitats. For example, natural recovery, closing mountains, and eliminating human disturbance can be considered for slightly degraded habitats. Natural regeneration and artificial restoration, such as planting seedlings and bamboo, may be suitable for moderately degraded habitats. Restoration involving extensive planting of seedlings and bamboo may be required for heavily degraded habitats. For low-quality habitats (i.e., artificial pure forests with single tree species), community transformation, such as thinning and planting of native tree species and bamboo, can be considered to improve the suitability [21,58]. Importantly, giant panda habitat restoration must consider the habitat requirements of this species.

## 5. Conclusions

This study analyzed the population size–habitat area relationship of giant pandas and found a linear relationship between lnS and lnA. A habitat area of 557.43 km^2^ was required to ensure the sustainable survival of a population with 25 pandas. This study selected 20 counties and 16 reserves containing ≥25 pandas. Six counties and 14 reserves had habitat areas smaller than 557.43 km^2^ and did not meet the minimum area requirements. The habitat area remained stable in the counties but decreased in the reserves between the third and fourth panda surveys. More habitat restoration is required in counties and reserves with a habitat area <557.43 km^2^ to increase the habitat. The reason for habitat decline should be determined in counties and reserves with a habitat area ≥557.43 km^2^ to implement targeted conservation measures. More long-term studies on local populations and habitat patches using remote sensing or incorporating genetic studies should be conducted to obtain accurate information for giant panda protection.

## Figures and Tables

**Table 1:** Linear model of the logarithm of population size (lnS) (logarithmic transformation of the population size) built by the logarithm of habitat area (lnA) (logarithmic transformation of the habitat area) of giant pandas.

Model	Non-Standardized Coefficient (B)	Standard Error	*t*-Value	*p*-Value	95% Confidence Interval of B
lnA	1.145	0.065	17.616	0.000	[1.012, 1.277]
Constant	−4.022	0.385	−10.436	0.000	[−4.808, −3.236]

**Table 2:** Tests for habitat area in the 20 counties and 14 nature reserves (using paired-sample *t*-test) from the third (1999–2003) to the fourth (2011–2014) giant panda survey.

Habitat Area	Mean (SD)		*t* Value	*p* Value
	Third Survey	Fourth Survey		
County (km^2^) (*n* = 20)	913.74 (643.35)	957.40 (617.04)	−1.575	0.132
Nature reserve (km^2^) (*n* = 14)	435.80 (326.55)	417.62 (258.83)	0.728	0.479

## Data Availability

Data are contained within this article and its Supplementary Materials.

## Supplementary Materials

The following supporting information can be downloaded at https://www.mdpi.com/article/10.3390/ani15020117/s1: Table S1: Local populations and habitat area of giant pandas [9]; Table S2: Number of giant pandas and habitat area in the 20 counties [9,11]; Table S3: Number of giant pandas and habitat area in the 16 nature reserves [9,11].

## References

[1] Hu J., Zhang Z., Wei F. (2011). History current situation and prospects on nature reserves for giant pandas (*Ailuropoda melanoleuca*) in China. Acta Theriol. Sin..

[2] Zhao X. (2006). The Giant Panda: Natural Heritage of Humanity.

[3] Wei F., Zhang Z., Hu J. (2011). Research advances and perspectives on the ecology of wild giant pandas. Acta Theriol. Sin..

[4] Jiang Z., Jiang J., Wang Y., Zhang E., Zhang Y., Li L., Xie F., Cai B., Cao L., Zheng G. (2016). Red list of China’s vertebrates. Biodivers. Sci..

[5] Swaisgood R., Wang D., Wei F. *Ailuropoda melanoleuca*. The IUCN Red List of Threatened Species 2016: eT712A121745669. https://www.iucnredlist.org/species/712/121745669.

[6] Kang D., Li J. (2016). Premature downgrade of panda’s status. Science.

[7] Xu W., Viña A., Kong L., Pimm S.L., Zhang J., Yang W., Xiao Y., Zhang L., Chen X., Liu J. (2017). Reassessing the conservation status of the giant panda using remote sensing. Nature Ecol. Evol..

[8] Hu J., Schaller G.B., Pan W., Zhu J. (1985). The Giant Pandas of Wolong.

[9] National Forestry and Grassland Administration (2021). The 4th National Survey Report on Giant Panda in China.

[10] Wang M.J., Li J.Q. (2008). Research on habitat restoration of giant panda after a grave disturbance of earthquake in Wanglang Nature Reserve, Sichuan Province. Acta Ecol. Sin..

[11] State Forestry Administration (2006). The 3rd National Survey Report on Giant Panda in China.

[12] Hull V., Zhang J., Zhou S., Huang J., Viña A., Liu W., Tuanmu M., Li R., Liu D., Xu W. (2014). Impact of livestock on giant pandas and their habitat. J. Nat. Conserv..

[13] Gong M.H., Ouyang Z.Y., Xu W.H., Song Y.L., Dai B. (2015). The location of wildlife corridors under the impact of road disturbance: Case study of a giant panda conservation corridor. Acta Ecol. Sin..

[14] Liu G., Gong M.H., Guan T.P., Chen L.M., Li H.X., Zhang Y., Zhou T.Y. (2016). A framework to evaluate impacts of tourism on giant pandas: A case study in Tangjiahe National Nature Reserve. Chin. J. Zool..

[15] Bearer S., Linderman M., Huang J., An L., He G., Liu J. (2008). Effects of fuelwood collection and timber harvesting on giant panda habitat use. Biol. Conserv..

[16] Ministry of Forestry, WWF (1989). A Comprehensive Survey Report on China’s Giant Panda and Its Habitat.

[17] Gong M., Yu C. (2003). Study on the Corridors of Giant Panda.

[18] Sichuan Forestry Department (2015). The Pandas of Sichuan: The 4th Survey Report on Giant Panda in Sichuan Province.

[19] Qing J., Xu C., Yang B., Yang Z., Qi D., Yang X., Gu X., Dai Q. (2016). Corridor design for the giant panda in the Xiaoxiangling Mountains. Acta Ecol. Sin..

[20] Wei F. (2018). Scientific Exploration of Wild Giant Panda.

[21] Li J., Shen G. (2012). The Habitat of Giant Pandas.

[22] Sichuan Precious Animal Resources Investigation Team (1977). Survey Report on Precious Animal Resources in Sichuan Province.

[23] Yan H., Xu Y., Zhao N., Yang S., Wang T. (2015). Medical Statistics.

[24] Zhang J. (2011). Quantitative Ecology.

[25] Backhaus K., Erichson B., Plinke W., Wang X., Weiber R. (2009). Multivariate Statistical Analysis: Using SPSS Tools.

[26] Pan W., Lv Z., Zhu X., Wang D., Wang H., Long Y., Fu D., Zhou X. (2001). Chance for Lasting Survival.

[27] Li J., Niu S., Liu Y. (2017). Forest Ecology.

[28] Li X.H., Li D.M., Yong Y.G., Zhang J. (1997). A preliminary analysis on population viability analysis for giant panda in Foping. Acta Zool. Sin..

[29] Ren W., Yang G., Wei F., Hu J. (2002). A simulation model for population viability analysis of giant panda in Mabiandafengding Nature Reserve. Acta Theriol. Sin..

[30] Zhang Z.J., Hu J.C., Wu H., Hou W.R. (2002). Analysis on population viability for giant panda in Tangjiahe. Acta Ecol. Sin..

[31] Zhang Z.J., Hu J.C. (2003). Population dynamics of the giant panda in the Daxiangling mountains according to PVA. J. Sichuan Teach. Coll. (Nat. Sci.).

[32] Zhu L., Wu P.W., Zhang H., Hu J.C. (2008). Population viability analysis of giant pandas in the Xiaoxiangling Mountains. J. China West Normal Univ. Nat. Sci..

[33] Jiang H.M., Hu J.C. (2010). Population viability analysis for the giant panda in Baoxing county, Sichuan. Sichuan J. Zool..

[34] Molles M.C. (2010). Ecology: Concept and Applications, 5th ed, Photocopies.

[35] Liu J., Linderman M., Ouyang Z., An L., Yang J., Zhang H. (2001). Ecological degradation in protected areas the case of Wolong nature reserve for giant pandas. Science.

[36] Liu X. (2010). 3S Technology and Wild Animal’s Habitat Assessment in China.

[37] Kang D., Li J. (2018). Role of nature reserves in giant panda protection. Environ. Sci. Pollut. Res..

[38] Loucks C.J., Lü Z., Dinerstein E., Wang H., Olson D.M., Zhu C., Wang D. (2001). Giant pandas in a changing landscape. Science.

[39] Liu X., Toxopeus A.G., Skidmore A.K., Shao X., Dang G., Wang T., Prins H.H.T. (2005). Giant panda habitat selection in Foping Nature Reserve, China. J. Wildl. Manag..

[40] Wei W., Swaisgood R., Dai Q., Yang Z., Yuan S., Owen M., Pilfold N., Yang X., Gu X., Zhou H. (2018). Giant panda distributional and habitat-use shifts in a changing landscape. Conserv. Lett..

[41] Ouyang Z.Y., Xu W.H., Wang X.Z., Wang W.J., Dong R.C., Zheng H., Li D.H., Li Z.Q., Zhang H.F., Zhuang C.W. (2008). Impact assessment of Wenchuan earthquake on ecosystems. Acta Ecol. Sin..

[42] Shen G.Z., Xie Z.Q., Feng C.Y., Xu W.T., Guo K. (2008). Influence of the Wenchuan earthquake on giant panda habitats and strategies for restoration. J. Plant Ecol. (Chin. Ver.).

[43] Xu X.L., Jiang D., Zhuang D.F., Qiu D.S. (2008). Assessment about the impact of Wenchuan earthquake on ecological environment. Acta Ecol. Sin..

[44] Xu W., Dong R., Wang X., Ouyang Z., Li Z., Xiao Y., Zhang J. (2009). Impact of China’s May 12 earthquake on giant panda habitat in Wenchuan County. J. Appl. Remote Sens..

[45] Ouyang Z.Y., Li Z.X., Liu J.G., An L., Zhang H.M., Tan Y.C., Zhou S.Q. (2002). The recovery processes of giant panda habitat in Wolong Nature Reserve, Sichuan China. Acta Ecol. Sin..

[46] Chen X., Wang X., Li J., Kang D. (2020). Species diversity of primary and secondary forests in Wanglang Nature Reserve. Glob. Ecol. Conserv..

[47] Chen X., Wang X., Li J., Kang D. (2021). Integrating livestock grazing and sympatric takin to evaluate the habitat suitability of giant panda in the Wanglang Nature Reserve. Animals.

[48] Zhang J., Hull V., Huang J., Yang W., Zhou S., Xu W., Huang Y., Ouyang Z., Zhang H., Liu J. (2014). Natural recovery and restoration in giant panda habitat after the Wenchuan earthquake. For. Ecol. Manag..

[49] Baishuijiang National Nature Reserve Administration. http://www.baishuijiang.com.cn/ms-mcms/html/1/2/85/9482.html.

[50] Qu G.P., Can M.Y., Zhao J.X., Tian L.H. (2016). Responses of community characteristics, soil carbon and nitrogen to different grazing management in Tibet alpine wetlands. Grassl. Turf.

[51] Sai J.M., Wang L., Wei J.J., Yang Y., Yang L.M., Mou P. (2021). The impacts of forest grazing on forest shrub-herbaceous layer. Environ. Ecol..

[52] Yang C.C., Chen K., Zhou Y.L., Chaoluomeng H., Chen Y. (2021). Effect of grazing on community characteristics and productivity of Xilingoul meadow steppe. Chin. J. Grassl..

[53] Zhang Z., Yuan S., Qi D., Zhang M. (2014). The Lushan earthquake and the giant panda: Impacts and conservation. Integr. Zool..

[54] Zhang J.D., Li Y.j., Huang J.Y. (2015). Advances in researches into influences of the earthquake on wild giant pandas. J. Sichuan For. Sci. Technol..

[55] Shen G.Z., Feng C.Y., Xie Z.Q., Ouyang Z.Y., Li J.Q., Pascal M. (2008). Proposed conservation landscape for giant pandas in the Minshan mountains, China. Conserv. Biol..

[56] Li S., Feng J., Li B.V., Lü Z. (2021). The Giant Panda National Park: Experiences and lessons learned from the pilot. Biodivers. Sci..

[57] Kang D. (2022). A review of the habitat restoration of giant pandas from 2012 to 2021: Research topics and advances. Sci. Total Environ..

[58] Chen D., Jin X., Zhang X., Zhu Q., Zhang Z., Hu S., Chen Y., Zhao Q. (2023). Giant panda habitat restoration requires more than just planting bamboo and trees. Restor. Ecol..

