# Supplementary material for: Relationship Between Population Size and Habitat Area of Giant Pandas in China
Source: Animals (Basel). 2025 Jan 7;15(2):117. doi: 10.3390/ani15020117 (PMC11758671; doi:10.3390/ani15020117)
Supplement: Supplementary file 1 [file animals-15-00117-s001.zip › animals-3375335-supplementary.pdf]

**Supplementary Materials**

**Table S1:** Local populations and habitat area of giant pandas [9].

**Table S2:** Number of giant pandas and habitat area in the 20 counties [9,11].

**Table S3:** Number of giant pandas and habitat area in the 16 nature reserves [9,11].

**Table S1:** Local populations and habitat area of giant pandas [9].

| Number | Name | Population size | Habitat area/$km^2$ |
|---|---|---|---|
| 1 | Qinling A | 7 | 211.41 |
| 2 | Qinling B | 20 | 594.22 |
| 3 | Qinling C | 277 | 2053.59 |
| 4 | Qinling D | 36 | 641.01 |
| 5 | Qinling E | 3 | 90.47 |
| 6 | Qinling F | 4 | 128.45 |
| 7 | Minshan A | 4 | 135.93 |
| 8 | Minshan B | 9 | 238.81 |
| 9 | Minshan C | 3 | 204.89 |
| 10 | Minshan D | 1 | 56.00 |
| 11 | Minshan E | 2 | 93.39 |
| 12 | Minshan F | 2 | 36.07 |
| 13 | Minshan G | 228 | 2227.97 |
| 14 | Minshan H | 2 | 34.06 |
| 15 | Minshan I | 1 | 33.69 |
| 16 | Minshan J | 167 | 2089.12 |
| 17 | Minshan K | 343 | 3199.26 |
| 18 | Minshan L | 35 | 1363.99 |
| 19 | Qionglaishan A | 92 | 943.76 |
| 20 | Qionglaishan B | 224 | 2558.33 |
| 21 | Qionglaishan C | 182 | 2567.79 |
| 22 | Qionglaishan D | 29 | 800.44 |
| 23 | Qionglaishan E | 1 | 17.27 |
| 24 | Daxiangling A | 4 | 205.75 |
| 25 | Daxiangling B | 32 | 979.16 |
| 26 | Daxiangling C | 2 | 43.78 |
| 27 | Xiaoxiangling A | 21 | 442.31 |
| 28 | Xiaoxiangling B | 9 | 751.33 |
| 29 | Liangshan A | 92 | 2165.97 |
| 30 | Liangshan B | 22 | 528.41 |
| 31 | Liangshan C | 3 | 173.60 |
| 32 | Liangshan D | 4 | 107.59 |
| 33 | Liangshan E | 3 | 48.12 |

**Table S2:** Number of giant pandas and habitat area in the 20 counties [9,11].

| Number | Province | County | Number of giant pandas in the fourth survey | Habitat area in the fourth survey/km$^2$ | Habitat area in the third survey/km$^2$ | Habitat area change rate/% |
|---|---|---|---|---|---|---|
| 1 | Sichuan | Pingwu | 335 | 2883.22 | 2770.71 | 4.1 |
| 2 | Sichuan | Baoxing | 181 | 1928.24 | 2000.32 | -3.6 |
| 3 | Sichuan | Wenchuan | 165 | 1483.39 | 1665.82 | -11.0 |
| 4 | Sichuan | Songpan | 111 | 1003.03 | 1113.61 | -9.9 |
| 5 | Sichuan | Tianquan | 78 | 1426.33 | 1308.35 | 9.0 |
| 6 | Sichuan | Beichuan | 74 | 956.91 | 889.51 | 7.6 |
| 7 | Sichuan | Ebian | 54 | 1130.57 | 942.99 | 19.9 |
| 8 | Sichuan | Qingchuan | 50 | 510.02 | 322.56 | 58.1 |
| 9 | Sichuan | Maoxian | 35 | 373.88 | 365.20 | 2.4 |
| 10 | Sichuan | Jiuzhaigou | 31 | 941.78 | 1062.75 | -11.4 |
| 11 | Sichuan | Lushan | 28 | 556.81 | 463.70 | 20.1 |
| 12 | Sichuan | Yingjing | 28 | 854.32 | 612.65 | 39.4 |
| 13 | Sichuan | Chongzhou | 26 | 258.98 | 195.84 | 32.2 |
| 14 | Sichuan | Dayi | 26 | 371.51 | 295.79 | 25.6 |
| 15 | Sichuan | Shimian | 25 | 660.06 | 436.94 | 51.1 |
| 16 | Shaanxi | Taibai | 102 | 933.75 | 796.79 | 17.2 |
| 17 | Shaanxi | Foping | 85 | 649.77 | 609.59 | 6.6 |
| 18 | Shaanxi | Yangxian | 69 | 516.89 | 543.28 | -4.9 |
| 19 | Shaanxi | Zhouzhi | 56 | 617.43 | 677.16 | -8.8 |
| 20 | Gansu | Wenxian | 112 | 1091.18 | 1201.31 | -9.2 |

**Table S3:** Number of giant pandas and habitat area in the 16 nature reserves [9,11].

| Number | Province | Nature reserve | Number of giant pandas in the fourth survey | Habitat area in the fourth survey/km$^2$ | Habitat area in the third survey/km$^2$ | Habitat area change rate/% |
|---|---|---|---|---|---|---|
| 1 | Sichuan | Wolong | 104 | 904.58 | 1145.88 | -21.1 |
| 2 | Sichuan | Xuebaoding | 92 | 542.21 | 475.14 | 14.1 |
| 3 | Sichuan | Baiyang | 82 | 524.08 | 580.25 | -9.7 |
| 4 | Sichuan | Xiaohegou | 49 | 258.93 | 263.56 | -1.8 |
| 5 | Sichuan | Caopo | 48 | 334.10 | 368.77 | -9.4 |
| 6 | Sichuan | Xiaozhaizigou | 47 | 401.03 | 404.45 | -0.8 |
| 7 | Sichuan | Tangjiahe | 39 | 329.52 | 289.07 | 14.0 |
| 8 | Sichuan | Fengtongzhai | 37 | 329.00 | 309.36 | 6.3 |
| 9 | Sichuan | Baodinggou | 35 | 345.86 | 186.00 | 85.9 |
| 10 | Sichuan | Heizhugou | 29 | 293.59 | — | — |
| 11 | Sichuan | Wanglang | 28 | 147.27 | 197.01 | -25.2 |
| 12 | Shaanxi | Foping | 67 | 292.40 | 292.00 | 0.1 |
| 13 | Shaanxi | Changqing | 57 | 290.72 | 299.00 | -2.8 |
| 14 | Shaanxi | Huangbaiyuan | 38 | 218.65 | — | — |
| 15 | Shaanxi | Laoxiancheng | 26 | 126.11 | 125.57 | 0.4 |
| 16 | Gansu | Baishuijiang | 110 | 1020.86 | 1165.15 | -12.4 |
